# Pulp Capping and Treatment of Pulp Canals

**Published:** 1885-01

**Authors:** J. H. Beebee


ARTICLE VII.

### PULP-CAPPING AND TREATMENT OF PULP-CANALS.

BY J. H. BEEBEE, ROCHESTER, N. Y.

[Read before the N. Y. 7th and 8th District Dental Society, Oct, 1884.]

The old subject of pulp capping and treatment of empty nerve canals is well worn, and it is very difficult to advance in this direction anything new.

I will first describe my ideal of a pulp cap: It must be disinfectant, antiseptic, antiphlogistic, and a non-conductor of thermal changes; it must also fit the surface covered, accurately, and adhere to it; at the same time it must be absolutely unyielding.

For the purposes of disinfection, and as a destroyer of germs of tooth decay, regardless of the nature of the latter, I have found nothing that is more efficient than the old perfumery of the dental office—creosote.

I am aware that very many of our practitioners have entirely banished it from their offices; but where an article has proved itself a good and faithful servant, it certainly seems poor policy to abandon it for anything less thoroughly subjected to the test of use.

As an antiphlogistic and antiseptic remedy iodoform has given the best results. I have seen the most obstinate cases of alveolar abcess promptly succumb to its soothing influence, when everything else had failed: pulps in a high state of imflammation have knelt before it, and cavities so sensitive as to render excavation impossible have been rendered comparatively painless when this drug has been held in them for a time, beneath a temporary filling.

It will be remembered that one part of the definition of the pulp cap as given calls for a non-conductor of thermal changes. The disastrous effects produced by the action of heat and cold upon the pulp in close contiguity to a metallic substance is too well known to all of us. Perhaps the best non-conductors are those of a vitreous or glassy nature. If an attempt is made to melt a piece of any of the resins it will be observed that while the surface of the piece is liquified the inner portions will remain brittle and hard, showing that this form of force is not readily conveyed by the atoms of the material. Among the hardest of the resins we find gum copal; it possesses the desirable property of solubility in one of the most volatile of substances—sulphuric ether. Another substance highly possessed of the property of resisting changes of temperature, is the phosphate or oxychloride of zinc, especially the former.

It is, perhaps, needless to say that no pulp should be capped while in a state of imflammation, it must, if diseased, show a great readiness to return to health, or give good evidence of healthy condition before the operation is performed. Having carefully removed all extraneous and decayed matter, unless a small amount of the latter be left immediately over the point of exposure, the cavity should be thoroughly saturated with kreosote, and wiped dry; then a saturated solution of iodoform in sulphuric ether should be introduced, and dried by permitting the solvent to evaporate. This is followed by copal ether varnish, of a syrup consistency, which is to be dried thoroughly with the aid of a hot air syringe. If the point of exposure is large, it is my custom, before the varnish is dry, to introduce a piece of thin linen writing paper, eut the shape of the floor of the cavity, which is to be pressed down with an instrument until it adheres in every part; then I re-varnish, and dry as before. Over the whole is to be applied and allowed to harden, a thinly mixed paste of oxyphosphate of zinc. In case of large exposures or diseased pulps, it is best, of course, not to finish with metal at the same sitting, the operator using his judgment as to completely filling the cavity with oxyphosphate or gutta percha in the case of temporary completion.

The great value of the varnish is, of course, its non-ccnducting property, and the certainty with which, without pressure, it will adapt itself to the surface covered. But another and very great benefit is the protection of the sensitive tissue from irritant action of the phosphoric acid in the plastic material, and especially is this the case when the paper before mentioned is used.

Have we not in the above that which we most desire—a cap that seals hermetically : that is a non-conductor of heat; that is rigid, and at the same time an arrester of decay, and soothing to the pulp, and preventive of all inflammatory action ?

The same remedial rgents are used in the treatment of pulp canals, and with satisfactory results. As before intimated, the most obstinate cases of alveolar abscess, either hidden at the apex of the root, or discharging through a fistula, rapidly improve when iodoform is presented to them. When all diseased action beyond the apical foramen has been removed, or is under control—and even sometimes when it is not, if there is a fistula for discharge, and the health of the patient is good, the canal should be thoroughly cleansed and dressed. It is not necessary to enlarge the canal unless it be extremely small, and it is desired to use a screw or other appliance for retaining a filling or crown. When cleanliness is ensured, apply kreosote as in pulp-capping, wiping thoroughly with cotton, wrapped in a nerve instrument. Next apply the ethereal solution of iodoform, and dry thoroughly ; now wet with alcohol, and having preciously cut some slim pointed pieces of gutta percha, force them one at a time into the ranal till it is filled. The alcohol will act as a lubricant, and will be forced out of the way, and in a short time the gutta percha will assume a consistency almost as firm as though it were solid. As I write, the thought has struck me that, perhaps, it might be well to use a thin sandrach varnish in place of the alcohol, and that would certainly make a solid and perfect plug*

*Since the above was read, the wriler has repeatedly used the varnish with the best of results.

Many practitioners prefer some of the various forms of the zinc plastics for filling the canal, but the experience of the writer is against them, as he has found that where pus comes in contact with such material, the plug is dissolved and a passage will be burrowed right through it. Then, too, they are not perfectly dense and non-absorbent substances. Gutta percha, however, is one of the best substances that can be used in the mouth, for it is able to resist the action of any and every fluid, be they corrosive or otherwise.

If I have presented anything that is interesting I am gratified; if nothing of interest, I thank you for your forbearance in listening to a short paper on a trite but hackneyed subject.—*Odontographic Journal.*

